# Student Perceptions of Narrative Feedback in Entrustable Professional Activities

**DOI:** 10.1111/tct.70089

**Published:** 2025-04-06

**Authors:** Rebecca Lee, Neil Dhami, William Gibson, Deena M. Hamza, Anna E. Oswald, Mandy Moffat

**Affiliations:** ^1^ Department of Medicine University of Alberta Hospital Edmonton Alberta Canada; ^2^ Centre for Medical Education, School of Medicine University of Dundee Dundee UK

**Keywords:** entrustable professional feedback, EPAs, narrative feedback, undergraduate medical education

## Abstract

**Introduction:**

Entrustable professional activity (EPA) observations can be used to develop a holistic picture of trainee competency in professional tasks. Narrative feedback is an essential component of EPAs, but there is a lack of published literature capturing undergraduate student perceptions.

**Methods:**

Students who completed Year 3 of the MD programme in 2022–2023 at one institution in Canada were invited to participate in a survey designed to elicit their perceptions of narrative feedback in EPAs. Survey methods included closed‐ended questions (analysed descriptively) and open‐ended questions (analysed through thematic analysis).

**Results:**

The response rate to the survey was 23%. Over 60% of students reported that narrative comments in EPAs were specific and aligned with EPA scores, and 86% reported that the narrative feedback was individualised at least some of the time. However, 57% reported that they never or rarely received actionable feedback for growth. Students demonstrated mixed feelings as to whether EPAs help support their clinical development. Some noted that they can help students identify gaps and reinforce positives. Others reported preferring verbal feedback to written feedback and that EPAs resulted in an administrative burden. Over 90% of students reported barriers to obtaining EPAs, and almost 90% expressed the need for changes to EPAs. A predominant theme from students was the desire for a reduction in EPA requirements.

**Conclusions:**

Students perceived the narrative feedback to be individualized and specific but reported that the feedback rarely contained feedback for growth. Students identified several barriers to EPA completion and provided recommended changes.

## 1 Introduction

Competency‐based medical education (CBME) is the foundation of postgraduate and undergraduate medical training in Canada [1]. Entrustable professional activities (EPAs) are a central concept within CBME and play an important role in providing clear, observable and assessable milestones in a trainee's progression. EPAs refer to specific tasks or responsibilities that trainees can be entrusted to do independently once they have demonstrated competency [2]. The focus on entrustment aligns with real‐world professional responsibilities, ensuring that trainees are prepared to meet the demands of independent practice [2].

The Association of Faculties of Medicine of Canada created 12 core EPAs in which all graduating medical students in Canada are expected to demonstrate competence (Appendix S1) [3]. These 12 EPAs were implemented at this institution in 2021. Faculty development on EPAs included synchronous and asynchronous opportunities, including information handouts, prerecorded videos and grand round sessions. Strategies to engage students included peer learning through student representatives, asynchronous videos and information handouts.

The narrative feedback captured in EPA observations serves multiple purposes. It is intended to justify the score given by faculty whilst also providing formative feedback to students via low‐stakes assessments [4]. It can advance learning outcomes by helping students reflect on their strengths and areas for growth [5].

Hattie and Timperley [6] conceptualised feedback as ‘information provided by an agent regarding aspect's of one's performance’. This model outlines three questions that need to be addressed in order to provide effective feedback: ‘where am I going?’ (feed up), ‘how am I going’ (feedback) and ‘where to next’ (feed forward) [6]. Feedback should include information on the student's goals, their progress in relation to these goals and feedback on actions that could reduce the gap between the student's current performance and their goals. Unfortunately, narrative feedback in EPAs rarely contains all three types of feedback [7].

Student views of feedback support the Hattie and Timperley feedback model. They would like specific feedback on what to improve on and, equally important, specific feedback on *how* to improve [8]. The quality of feedback may influence student perceptions where lower quality feedback is perceived as less valuable.

There is a paucity of evidence about undergraduate student perceptions of narrative feedback in EPA observations. Trainees in postgraduate medical education report that EPAs have increased the quantity of feedback whilst failing to improve the quality [9]. There is the possibility that student perceptions are similar; however, students generally have less autonomy than postgraduate trainees, influencing their perceptions of feedback [10]. It has also been demonstrated that junior learners value positive feedback whereas more senior learners value specific and constructive feedback [11].

The purpose of this study was to explore undergraduate medical student perceptions of narrative feedback in EPA observations. Specifically, this study aimed to explore whether narrative feedback in EPA observations is perceived by students as helpful for their clinical development, whether students perceive any barriers to obtaining EPA observations and how students feel that use of EPA observations could be improved to support learning.

The purpose of this study was to explore undergraduate medical student perceptions of narrative feedback in EPA observations.

## 2 Materials and Methods

### 2.1 Participants

Students who completed Year 3 of the MD programme in 2022–2023 at one institution in Canada were invited via email to participate in an online survey designed to elicit their perceptions of narrative feedback in EPA observations.

Through the online survey format, information about the study was provided, followed by an implied consent statement. Participants could withdraw consent at any time during the survey by exiting the online form prior to submission. Once submitted, survey responses could not be withdrawn, as data were collected anonymously.

### 2.2 Data Capture

The survey included a combination of closed and open‐ended questions and was distributed via REDCap, a secure web platform for building and managing surveys, which has been shown to be easy to use and reliable [12]. The survey contained 5‐point Likert questions and free‐text response questions (Appendix S2). Different question types were chosen because student perceptions are multifaceted and therefore are best captured using varied data collection.

All data were encrypted and stored on the institution's secure network. Only the study team had access to the data. Ethical approval was obtained from the institutional Research Ethics Board and Trainee Research Access Committee.

### 2.3 Data Analyses

Descriptive statistics were used to analyse closed ended questions, such as the median and mode [13]. Excel was used to manage the quantitative data.

Thematic analysis, a process where themes from narrative data are identified and analysed, was used to synthesise narrative comments in the survey [14]. An inductive approach was utilised to allow the survey data to determine the themes [15]. Microsoft Word was used to store and manage the qualitative data. Two members of the research team (ND and RL) performed the thematic analysis. All of the narrative comments provided by students to the free‐text questions in the survey were reviewed to familiarise the investigators with the data. Initial codes were then generated. Themes were then identified and reviewed to ensure they were complete and meaningful and to ensure that they were sufficiently different to warrant separate themes. Operational saturation was achieved when no new codes or themes were able to be generated. The themes were then named and the key components of each theme elaborated on. Disagreements were resolved through consensus discussion.

## 3 Results

### 3.1 Closed Ended Survey Questions

**FIGURE 1:**
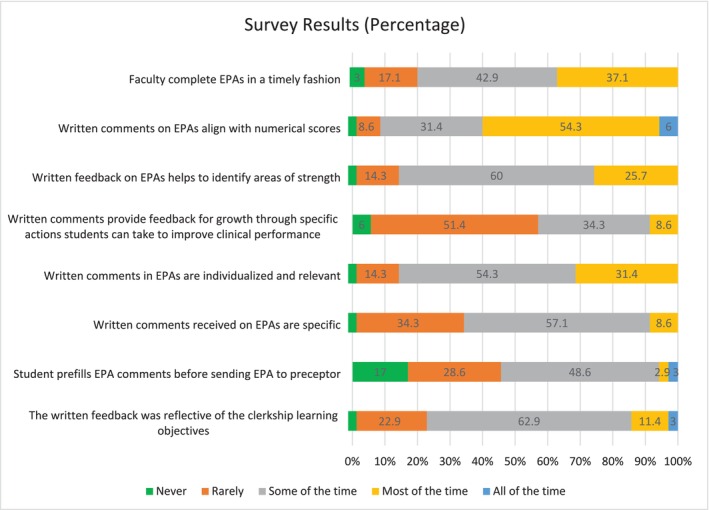
Survey results: Part 1.

**FIGURE 2:**
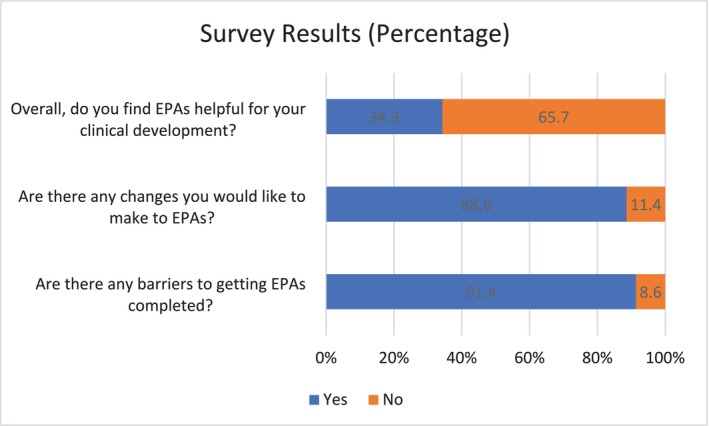
Survey results: Part 2.

In total, 35 out of 153 students (23%) completed the survey. The results of the closed ended questions are shown below in Figures 1 and 2.

The survey results were allocated a numerical value, with *never* being represented as ‘1’ and *all of the time* being represented as ‘5’. The median was 3, and the mode was 3 for all questions with the exception of the question on specific actions included in narrative comments, which had a median of 2 and a mode of 2, and the question on narrative comment alignment with numerical scores, which had a median of 4 and a mode of 4.

The majority of students, 86%, reported that the narrative feedback in EPA observations was individualised some or most of the time. Over 60% reported that narrative comments were aligned with EPA scores most or all of the time. Sixty per cent reported that it helped them to identify areas of strength some of the time. However, 20% reported that faculty rarely or never complete EPA observations in a timely manner, and 57% reported that they rarely or never receive feedback for growth.

The majority of students, 86%, reported that the narrative feedback in EPA observations was individualised.

### 3.2 Open‐Ended Survey Questions

#### 3.2.1 Survey Domain No. 1: Are EPA Observations Helpful for Clinical Development?

The overall theme for this domain was that students have varying perceptions about whether EPA observations are helpful for their clinical development. Students who found the narrative feedback helpful noted that gaps in their learning were made clear. Students who did not find EPAs helpful noted that immediate verbal feedback obtained in daily clinical activities was more helpful and that narrative feedback was redundant following this. Finally, some felt that EPAs were being used to force the programme to make improvements to supervision levels. Representative quotes for this theme are shown in Table 1.

**TABLE 1:** Are EPA observations helpful for clinical development?

Survey Domain No. 1: Are EPA observations helpful for clinical development?	
Category: Helpfulness of Narrative Feedback	
Overall theme: There was variability in student perceptions on the helpfulness of narrative feedback in EPA observations	
Subtheme	Representative quotes
Helpful for learning	‘It pushes me and colleagues to reflect on our performance, strengths, and weaknesses more often than we otherwise would’. ‘EPAs are absolutely valuable—I know some colleagues will say that they feel worthless or like an unnecessary hurdle, but I think that's more to do with some of the misuse of EPAs rather than what they are fundamentally designed to do and how they can indeed be useful feedback tools. Even though some students report them as just a checklist of frustrating items to collect, I think many students have expressed the value of feeling supported and taught well based on specific actionable feedback in an EPA (especially those done by residents who often put in more detail) and that is a core benefit that I would not want to sacrifice’. ‘I think when there is time to fill them out properly they can be helpful to identify gaps and reinforce positives’.
An unhelpful burden	‘I often found this (EPAs) a distraction from my clinical learning and duties. I was constantly stressed about obtaining the required EPAs and would be focused on checking boxes rather than being involved in patient care, reading around cases, and applying practical feedback. EPAs felt forced and tedious for both students and preceptors’. ‘I get verbal feedback from supervisors and residents regardless of EPAs, EPAs are just a formality that are in the way and are in no way useful to myself. The responses are used by the school to see how we progress but as a student, I never read them, reflect on them, or even learn from them. If anything they make my rotations more stressful and more work, and even if I try my best to follow up and have staff complete them ‐ sometimes they just will not and then the administrators come after ME for not having it done’. ‘Immediate, specific, verbal feedback is much more useful’.
Role in programme development	‘I think that EPAs are used to make low quality programs better and force programs that lack hands‐on learning to prioritize this. However, programs that are already great, suffer from EPAs and take away from actual learning and force more work and administrative duties to the student and the staff. I literally have nothing positive to say about EPAs’. ‘I sometimes wonder if they are actually present to force staff to provide adequate supervision and education for students because if I did not need an EPA in some of my clinical rotations, the staff would never have supervised me doing things’.

Students have varying perceptions about whether EPA observations are helpful for their clinical development.

#### 3.2.2 Survey Domain No. 2: Barriers to Obtaining EPA Observations

The overall theme in this domain was that there were barriers to students obtaining EPA observations. Students identified that it could be challenging to get staff to complete EPA observations, with many staff refusing or agreeing, but subsequently failing to complete them. Students reported feeling uncomfortable when requesting EPA observations due to awareness that faculty are busy. A final subtheme identified was that there can be challenges obtaining specific EPAs, particularly EPAs, which require direct observation. Representative quotes for this theme are shown in Table 2.

**TABLE 2:** Barriers to obtaining EPA observations.

Survey Domain No. 2: barriers to obtaining EPA observations	
Category: barriers to EPA observations	
Overall theme: Students reported multiple barriers to obtaining EPA observations	
Subtheme	Representative quotes
Challenges in getting staff to complete EPA observations	‘Preceptors refusing to complete them or agreeing to (complete EPAs) then ignoring any and all reminders and having them expire’. ‘… on some services …, I rarely worked directly with staff (spent 90% of my time with residents) so it was difficult to obtain the necessary staff EPAs’.
Student discomfort at requesting EPA observations	‘Staff rarely offer and usually seem too busy so it is too intimidating to ask for them’. ‘Staff are often very busy and this makes it feel like I am asking them for a favour every time I ask for one. This limits the amount I ask for’. ‘EPAs are a huge inconvenience to staff and residents so I feel uncomfortable to ask EPAs of people especially since I need so many’.
Challenges obtaining specific EPA observations	‘Staff have virtually never observed my histories or physicals so making me get an EPA for this every block is extremely annoying and unrealistic—staff will never do this’. ‘Depending on the rotation it can sometimes be hard to get a clinical experience relevant for the EPAs (e.g. an observed history is difficult to get as it seems to disrupt team flow if the preceptor has to sit and watch the medical student doing a history, especially on busy rotations)’. ‘EPAs also have very particular requirements which can make it difficult to get on rotations where we have little opportunity and responsibility … On some rotations, it is difficult to get observed assessments or even have the opportunity to participate in order to be evaluated’.

#### 3.2.3 Survey Domain No. 3: Changes to EPA Observations

The overall theme in this domain was that students felt that there should be changes made to EPA observations. The predominant subtheme was that students felt that there should be a reduction in EPA requirements with an increased emphasis on more global evaluations of performance to permit students to focus on clinical learning and to reduce EPA burden. A further subtheme that emerged was moving the onus for completing the EPA observations from students to preceptors so that students are not penalised for staff not completing them. A final subtheme was changing the wording on EPA observation forms to make the criteria more explicit as students felt that staff often misunderstood the scoring scale. Representative quotes for this theme are shown in Table 3.

**TABLE 3:** Changes to EPA observations.

Survey Domain No. 3: changes to EPA observations	
Category: changes to EPA observations	
Overall theme: Students recommended changes to EPA observations	
Subtheme	Representative quotes
Reduction in EPA observation requirements	‘Less volume of EPAs required. Often times this disrupts learning as I am focused on trying to get a specific EPA rather than taking advantage of other learning opportunities or being present with the patient in a visit’. ‘Less EPAs, more pre‐filled feedback to decrease feedback fatigue’.
More emphasis on global evaluations of performance	**‘**When working with one preceptor (and no residents) for several weeks …, it would be helpful to have less EPAs to complete as we are already obtaining feedback regularly through clerkship evaluations at the midpoint and final of the rotations. Would help with feedback fatigue for staff and students’. **‘**I think like electives, we should send overall weekly evaluations rather than EPAs around a specific case or skill. Keeps us as students more accountable too to continue showing up and performing all week. Would also allow for better feedback I think rather than having to always ask for so many per rotation’. **‘**Global performance assessments seem to be better rather than feedback on specific encounters. Being scored on a single encounter does not provide opportunities to have comments on overall progress /strengths/deficits. As a student I choose encounters that I felt went really well for these types of EPAs when a single excellent encounter (or a really bad one) is not representative of my overall performance’.
Moving the onus of EPA observation completion to preceptors	‘Making them mandatory for staff to complete rather than for students to have them completed’. ‘More compensation or compelling reasons for staff to complete EPAs so that we do not have to feel so guilty for requesting their precious time to fill them out, given that they are mandatory for us’. ‘Less onus on students to have staff complete them in a timely manner (i.e. if multiple reminders sent and still not completed it should not be used against the student/towards the minimum required for the rotation)’.
Changing the wording of EPA observation forms	‘Change the wording from “assessing as first year resident” as many preceptors are under the impression they can never select the “functioning at this level” box as we are not first year residents. This is then turned into a potentially failing numerical score’. ‘I would like EPAs to include explicit expectations for preceptors to understand what each category is meant to represent, so that subjective variability in “what's good enough for first day of residency” is not deemed to be “well I cannot give you full marks because you have still gotta get better later.”’ ‘I think the number basis at the top is not helpful given its supposed to be formative feedback and most staff and residents click “functioning at a residents level” regardless. I also feel they are fairly non‐specific’.

## 4 Discussion

The majority (66%) of students report that EPAs may not contribute to their clinical development as intended, yet report positive impressions in several subcomponents. For example, the majority (77%) of students report that the narrative feedback was reflective of the clerkship learning objectives, which suggests that feedback is provided on the goals that students are working towards. The majority (86%) report that the feedback received helped to identify areas of strength and that the feedback was specific, individualised and relevant, at least some of the time. This suggests that they are receiving narrative feedback on their current performance. However, 57% of students report rarely or never receiving narrative comments on how to improve or work towards a goal.

Using Hattie and Timperley's feedback model, these results suggest that students are being provided with feedback on ‘where am I going’ and ‘how am I going’ most of the time but rarely on ‘where to next’. This is in keeping with published literature, which suggests that ‘feed forward’ feedback is the least frequently delivered despite the fact that this type of feedback has the greatest impact [6].

Students do not feel that faculty complete EPA observations in a timely fashion, with 20% reporting that faculty rarely or never complete them in a timely manner. This is concerning because evidence suggests that delayed feedback results in poorer quality feedback and is perceived as less credible [8].

### 4.1 Narrative Responses to Survey

Credibility of the qualitative data was established by ensuring investigator triangulation after independent and dual review of the data [16]. Year 4 students were intentionally selected as the study population to ensure that participants had experienced EPA observations [17]. The surveys were anonymous to eliminate potential confidentiality concerns, which could negatively impact on the trustworthiness of the data [17]. The findings from this study are similar to research involving residents, which indicate that these results are derived from data [9, 16]. Clear records of the research path ensured that the research is dependable and confirmable [16]. This study provides a detailed description of the participants and the research process to enable readers to assess for transferability to their settings [16].

### 4.2 Are EPA Observations Helpful for Clinical Development?

This study demonstrates that students have mixed feelings as to whether EPAs help support their clinical development. Some students report that EPA observations encourage them to reflect on their performance more often than they would without the inclusion of EPA observations in their learning. Self‐reflection is felt to be a key element of professional development, and it has been shown that increased reflection is associated with an improved learning experience [18].

Others felt that EPA observations forced faculty to provide supervision and feedback. This has been reported in postgraduate medical education literature but, to our knowledge, has not previously been reported in undergraduate medical education [19]. This raises the question of whether the required documentation of the EPA observations may be prompting and/or promoting the verbal feedback that learners report that they value and prefer.

### 4.3 Barriers to Obtaining EPA Observations

Over 90% of students report barriers to obtaining EPA observations, which likely adds to the perception that EPA observations are an administrative burden. Students report that faculty often refuse to complete them or initially agree but then fail to complete them. It is possible that students who perceive that EPA observations are an administrative burden are less likely to meaningfully engage with the feedback received in EPA observations, thus reducing learning opportunities.

Over 90% of students report barriers to obtaining EPA observations.

Faculty recognise that time to complete EPA observations is a barrier to completing them and that competing demands often mean that EPA observations are overlooked [20]. Students report being aware of faculty workload and, as a result, feel uncomfortable requesting EPA observations, limiting their ability to acquire them.

If undergraduate medical education wishes to continue using EPAs to drive learning and assess competencies, it is imperative that steps are taken to address faculty engagement and accountability. Lack of faculty engagement with EPA observations can negatively impact the quality of feedback provided to students and can increase learner anxiety around requesting EPA observations [20].

Difficulties obtaining EPA observations in certain clerkship settings provide an opportunity for programmes to reassess which EPA observations are required in each clerkship. Students in this survey suggested that some EPAs should only be assessed in certain clerkship settings due to lack of specific learning opportunities. Another interpretation is that difficulties obtaining EPA observations can be used to identify clerkships, which require development to create richer learning opportunities [21].

### 4.4 Changes to EPAs and EPA Observations

Almost 90% of students expressed the need for changes to be made to EPA observations. The predominant subtheme was a desire for a reduction in EPA observation requirements. Students felt that the volume of EPA observations hampered, rather than supported, learning and that EPA observations took away from other valuable learning opportunities. This raises the discussion of how many EPA observations should be required. As the number of observations decrease, they may be perceived as more high stakes events. To date, there are no clear data to suggest what the minimum number of EPA observations a student should obtain to be viewed as having achieved competence for a graduating medical student [22].

The predominant subtheme was a desire for a reduction in EPA observation requirements.

Some students felt that global performance assessments should be used instead of EPA observations. Some felt that global performance assessments provide more overall feedback and a more holistic assessment of student's strengths and weaknesses. Interestingly, one publication found that EPA observations discriminated clinical performance better than global clinical performance assessments [23]. However, another study reported that EPA observations and global assessment tools may capture different aspects of clinical performance, indicating that there may be a role for both [24].

Another subtheme that emerged was to move the onus of EPA observation completion from students to faculty. Some suggested financial compensation to incentivise completion. From a practical perspective, this is unlikely to be a realistic solution due to cost. In this institution, the number of EPA observations completed is reported and recognised in faculty's annual report. However, given the ongoing challenges that students report, it does not appear that this is having a significant impact on EPA observation completion.

The third subtheme identified involved the specific wording of EPA observation forms. Multiple students felt that the language on the scoring scale was poorly understood by staff. This aligns with other research findings, which report that the interpretation of the scale is dependent on the exact phrasing that the scale uses [25]. Students suggested that the EPA observation forms include specific expectations for each score to help reduce subjective variability.

### 4.5 Limitations

Only 23% of eligible students completed the survey; however, it has been found that surveys with smaller sample sizes only require a 20%–25% response rate to provide fairly confident estimates [26]. The response rate also aligns with response rates from other surveys of healthcare professionals, with reported average response rates for online surveys ranging from 13% to 46% [27, 28].

One concern with low response rates is whether the survey data are generalizable to nonrespondents. Most nonresponse to surveys is passive in nature where potential participants forget about the survey. Passive nonrespondents have been found to be attitudinally similar to survey respondents [29]. Conversely, active nonrespondents make a deliberate decision to not complete the survey. Active nonrespondents tend to differ attitudinally from respondents but only represent approximately 15% of nonrespondents [29]. As a result, we feel that the survey results are generalizable to nonrespondents.

Response rate is only one component of survey validity. By inviting all Year 4 students, we ensured that potential participants were representative of the population, which we hoped to study [17]. It also ensured that all participants were qualified to participate because they had all engaged with EPAs.

Another limitation of this study was this it was a single centre study; however, the transparency of the methods and study processes foster the ability for this work to be transferable to other institutions.

### 4.6 Implications

This study provides a valuable contribution to understanding student perceptions of narrative comments in EPA observations. Future research can build on these results to further develop understanding of this important issue.

Students reported the view that verbal feedback was more helpful than narrative comments in EPA observations. Future research could capture verbal feedback through audio recordings and assess the objective quality and student perceptions of this feedback. Future research could also study whether narrative feedback in EPA observations could be improved by using artificial intelligence to summarise verbal feedback provided by preceptors.

Faculty development has been suggested to improve faculty engagement with EPAs, but it is unclear what format this should take [30]. Future research should evaluate the impact of different formats of faculty development on both faculty engagement and on the quality of narrative comments provided to students.

Students expressed a wish for changes to be made to EPAs. Future research should focus on whether interventions such as changes to EPA requirements result in improved feedback quality and student satisfaction.

One final consideration is whether today's students will be more engaged with EPAs once they become faculty, given that they have experienced them from the student perspective. Alternatively, will they learn from supervisors to resent EPAs in an example of the hidden curriculum?

## 5 Conclusion

The results from this study suggest that students perceived the narrative feedback they received via EPA observations to be individualised, and specific. They perceived that the feedback they received never or rarely included feedback for growth.

Students identified several barriers to EPA observation completion and recommended several changes to be made, most prominently reducing the number of EPA observations required.

Finding balance between the number of EPA observations that students are required to complete whilst maintaining meaningful feedback and other benefits of EPA observations must be a key focus in the future for undergraduate medical programmes.

## Author Contributions

**Rebecca Lee:** conceptualization, methodology, investigation, formal analysis, resources, project administration, writing – review and editing, writing – original draft. **Neil Dhami:** formal analysis, writing – review and editing. **William Gibson:** writing – review and editing. **Deena M. Hamza:** writing – review and editing, supervision. **Anna E. Oswald:** writing – review and editing, supervision, resources. **Mandy Moffat:** writing – review and editing, supervision.

## Ethics Statement

Ethical approval was obtained from the institutional Research Ethics Board and Trainee Research Access Committee (institutional requirement when requesting student data).

## Conflicts of Interest

The authors declare no conflicts of interest.

## Supporting information


**Appendix S1** Supporting Information.

## Data Availability

The data that support the findings of this study are available on request from the corresponding author (RL). The data are not publicly available due to containing information that could compromise the privacy of the research participants. There are not any other papers (in progress, under review or published) that use the same dataset.
