# Supplementary material for: Student Perceptions of Narrative Feedback in Entrustable Professional Activities
Source: Clin Teach. 2025 Apr 6;22(3):e70089. doi: 10.1111/tct.70089 (PMC11973025; doi:10.1111/tct.70089)
Supplement: Supplementary file 1 — Appendix S1 Supporting Information. [file TCT-22-e70089-s001.docx]

**Appendices**

**Appendix A**

| **Association of Faculties of Medicine of Canada Core EPAs** | |
| --- | --- |
| **EPA** | **Description** |
| EPA 1 | Obtain a history and perform a physical examination adapted to the patient’s clinical situation |
| EPA 2 | Formulate and justify a prioritized differential diagnosis |
| EPA 3 | Formulate an initial plan of investigation based on the diagnostic hypotheses |
| EPA 4 | Interpret and communicate results of common diagnostic and screening tests |
| EPA 5 | Formulate, communicate and implement management plans |
| EPA 6 | Present oral and written reports that document a clinical encounter |
| EPA 7 | Provide and receive the handover in transitions of care |
| EPA 8 | Recognize a patient requiring urgent or emergent care, provide initial management and seek help |
| EPA 9 | Communicate in difficult situations |
| EPA 10 | Contribute to a culture of safety and improvement |
| EPA 11 | Perform general procedures of a physician |
| EPA 12 | Educate patients on disease management, health promotion and preventive medicine |

Association of Faculties of Medicine of Canada, 2019

**Appendix B**

Student Survey:

1. The written feedback I receive on EPAs are reflective of the learning objectives of the clerkship:

- All of the time
- Most of the time
- Some of the time
- Rarely
- Never

1. I pre-fill the comments on the EPAs before sending the EPA to the preceptor (i.e. the preceptor does not provide their own written comments):

- All of the time
- Most of the time
- Some of the time
- Rarely
- Never

1. Written comments on EPAs can be general or specific. An example of general feedback would be “good job”. An example of specific feedback would be “I noticed that you identified that an adverse drug reaction was the likely cause of this patient’s presentation”.

Written comments I receive on EPAs are specific:

- All of the time
- Most of the time
- Some of the time
- Rarely
- Never

1. Written comments in EPAs are individualized and are relevant to me:

- All of the time
- Most of the time
- Some of the time
- Rarely
- Never

1. Written comments I receive on EPAs provide feedback for growth through specific actions I can take to improve my clinical performance:

- All of the time
- Most of the time
- Some of the time
- Rarely
- Never

1. Written feedback I receive on EPAs help me to identify areas of strength:

- All of the time
- Most of the time
- Some of the time
- Rarely
- Never

1. Written comments on EPAs I receive are aligned with numerical scores:

- All of the time
- Most of the time
- Some of the time
- Rarely
- Never

1. Faculty complete my EPAs in a timely fashion:

- All of the time
- Most of the time
- Some of the time
- Rarely
- Never

1. Are there any barriers to getting my EPAs completed?

- Yes
- No

Please provide details on any barriers –

1. Are there any changes you would like to make to EPAs?

- Yes
- No

Please provide details of any changes you would like to see to EPAs

1. Overall, do you find EPAs helpful for your clinical development?

- Yes
- No

Please provide comments to support your answer

1. Are there any other comments you would like to make about written comments in EPAs or EPAs in general?
